# The Incidence of Head Acceleration Events During Pitch‐Based Training and Match Play in Professional Men's Rugby League

**DOI:** 10.1111/sms.70156

**Published:** 2025-11-08

**Authors:** James Parmley, Dan Weaving, Sarah Whitehead, James Tooby, Cameron Owen, Thomas Sawczuk, Greg Roe, Neil Collins, Gemma Phillips, Dane Vishnubala, Keith Stokes, Sam Hudson, Ben Jones

**Affiliations:** ^1^ Centre for Applied Rugby Research (CARR) Centre, Carnegie School of Sport Leeds Beckett University Leeds UK; ^2^ Institute of Sport Manchester Metropolitan University Manchester UK; ^3^ Department of Physical Activity and Sport, Faculty of Arts and Sciences Edge Hill University Ormskirk UK; ^4^ Applied Sports Science and Exercise Testing Laboratory The University of Newcastle Ourimbah New South Wales Australia; ^5^ England Performance Unit Rugby Football League Manchester UK; ^6^ Obesity Institute Leeds Beckett University Leeds UK; ^7^ Uno‐X Mobility Oslo Norway; ^8^ Faculty of Biological Sciences School of Biomedical Sciences Leeds UK; ^9^ Clinical Exercise and Rehabilitation Research Centre, School of Human Sciences University of Derby Derby UK; ^10^ School of Public Health Imperial College London London UK; ^11^ Centre for Health, and Injury & Illness Prevention in Sport University of Bath Bath UK; ^12^ UK Collaborating Centre on Injury and Illness Prevention in Sport (UKCCIIS) University of Bath Bath UK; ^13^ Rugby Football Union Twickenham UK; ^14^ PREM Rugby London UK; ^15^ School of Behavioural and Health Sciences, Faculty of Health Sciences Australian Catholic University Brisbane Queensland Australia; ^16^ Division of Exercise Science and Sports Medicine, Department of Human Biology, Faculty of Health Sciences The University of Cape Town and the Sports Science Institute of South Africa Cape Town South Africa

**Keywords:** contact, head acceleration events, instrumented mouthguards, Rugby, training

## Abstract

This study aimed to describe the incidence of head acceleration events (HAEs) during pitch‐based in‐season training and matches in professional male rugby league. Data were recorded using instrumented mouthguards from 108 players (70 forwards and 38 backs) at nine Super League teams (2024 season), resulting in 468 player‐training sessions and 665 player‐matches included. Peak linear and angular acceleration were calculated from each HAE and analyzed using generalized linear mixed‐effects models. During the 468 player‐training sessions, 814 HAEs above the lowest magnitude threshold (5 *g* and 400 rad.s^−2^) were observed and the mean HAE incidence rate per player‐hour was 1.52 (95% confidence intervals; 1.34–1.70). This was substantially lower than matches (25.78 [23.28–28.27] per player‐hour) with HAE incidence being 17 times greater during matches compared to training (incidence rate ratio 16.96 [14.92–19.01]). Higher magnitude HAEs had a lower incidence in both training and matches (e.g., > 25 *g* 0.04 [0.02–0.06] and 2.01 [1.79–2.24] per player‐hour). Out of 468 player‐training sessions, 307 (~66%) had no HAEs > 10 *g* and 441 (~94%) had no HAEs > 25 *g*. Overall, the incidence rates of HAEs during training were low and substantially lower than match‐play. However, a small proportion of relatively high in magnitude HAEs do occur during training, which could be the target of prevention interventions in training. However, given the different HAE rates between training and matches, interventions targeting matches (e.g., law modifications or reduced exposure) would have a larger effect on reducing HAEs for players than training interventions.

## 1 Introduction

Rugby league players are exposed to contact events during both training and match play [1], which pose a potential risk of injury [2, 3, 4]. Contact events also result in head acceleration events (HAEs) [5, 6, 7, 8], which are reported to have potential associations with negative brain health outcomes later in life [9] and biomarkers of brain injury [10]. Given these concerns, the Rugby Football League (RFL; national governing body for rugby league in England) recently introduced multiple strategies to limit and monitor contact exposure to mitigate risk. These include the introduction of contact load guidelines to reduce the volume of contact training within the training week [11] the implementation of instrumented mouthguards (iMGs) to monitor HAEs [12, 13] and match limits to reduce HAEs [6].

During rugby league match play, iMGs have been used to quantify head kinematics, with HAE incidence rates being reported as between 0.86 and 2.20 per match for HAEs > 25 *g* magnitude [7]. Rugby league players spend more time in training than in matches [11]. Therefore, the rate of HAEs during training needs to be understood. Research in rugby union reported that HAE rates in training were lower than match play; players were between 6.88 and 7.02 times more likely to experience a HAE per hour of match play than training, dependent on playing position [14]. However, this is not known in rugby league. Therefore, the aim of this study was to quantify and describe the incidence of HAEs during pitch‐based training where contact training occurs and compare to match play in professional male rugby league players.

## 2 Methods

### 2.1 Study Design and Participants

**FIGURE 1:**
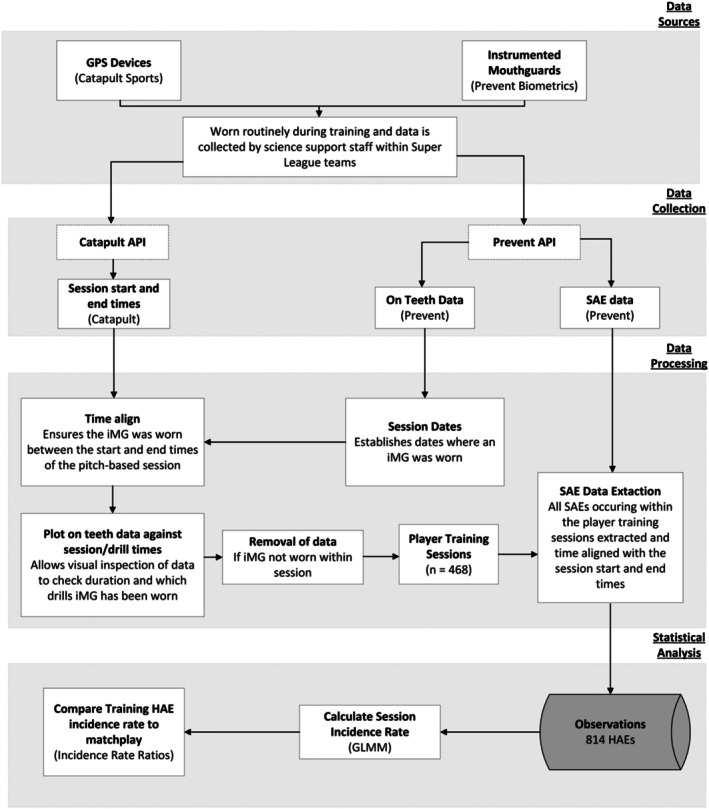
Workflow of procedures to collect, process and analyze training sensor acceleration event (SAE) data within the study.

An observational cohort design was conducted. All data collection occurred in training and matches during the 2024 Super League season, conducted as part of the RFL Tackle and Contact Kinematics, Loads and Exposure (TaCKLE) project. Data from GPS devices (Vector S7, Catapult Sports, Melbourne, Australia) were analyzed to determine training exposure, and iMGs (Prevent Biometrics, Minneapolis, MN, USA) to determine HAE exposure. Ethics approval (number: 100411) was granted by the Leeds Beckett University Ethics Committee. Participation was voluntary and all participants provided informed written consent. All 12 Super League teams were recruited to participate; however, three teams were unable to participate as they did not wear GPS devices (*n* = 1) or iMGs (*n* = 2) during training. Only players who wore iMGs during both training and matches were included in the study. Data from 108 male players (70 forwards and 38 backs) from 9 Super League teams were included. This led to a total of 468 player‐training sessions (median [interquartile range (IQR)] = 3 [1–5]; range = 1–29 observations per player) from 143 total team training sessions (median [IQR] = 11 [5–28]; range = 1–37 observations per team). There were 665 player matches (median [IQR] = 7 [3–12]; range = 1–25 observations per player) from 197 team matches (median [IQR] = 23 [21–25]; range = 14–37 observations per team). Figure 1 summarizes the data sources, collection, processing, and statistical analysis used within the study.

### 2.2 Data Sources

#### 2.2.1 Instrumented Mouthguards (iMGs) to Measure Head Acceleration Events

All players underwent 3D dental scans and were provided with custom‐fit iMGs (Prevent Biometrics, Minneapolis, MN, USA; Version 2.0), which were used to collect sensor acceleration event (SAE) data as a measure of HAEs, consistent with previous methods [7]. The iMGs house linear accelerometers and gyroscopes, with a 3200 Hz sampling rate, and measurement ranges of ±200 *g* and ±34.9 rad⋅s^−2^ [12]. The iMGs were configured with an event trigger threshold of 8 *g* along any axis of the accelerometer, which initiated the recording of an SAE. Each SAE consisted of 10 ms of pre‐trigger kinematic data and 40 ms of post‐trigger kinematics. Linear kinematics were transformed to the estimated centre of gravity (CoG). Angular acceleration values were not transformed based on the assumption of the head being a rigid body [14]. All linear and angular kinematic data from SAEs were initially processed by Prevent Biometrics using a four‐pole, zero‐phase Butterworth low‐pass filter with a 200 Hz cutoff frequency. Peak linear acceleration (PLA; *g*) and peak angular acceleration (PAA; rad⋅s^−2^) were extracted from resultant kinematics from each SAE. This study only includes SAEs exceeding both 5 *g* and 400 rad⋅s^−2^ to reduce the inclusion of SAEs triggered by noncontact events [15, 16]. Furthermore, only SAEs classified as true positives by Prevent Biometrics' in‐house event discrimination algorithm were used based on high positive predictive values in previous validations within rugby league [7, 12, 14]. A Prevent Biometrics algorithm categorized SAEs based on the level of noise in the signal as containing minimal (*n* = 691), moderate (*n* = 54), or severe (*n* = 69) noise in the training dataset and minimal (*n* = 16 461), moderate (*n* = 903), or severe (*n* = 679) noise in the match dataset. The iMGs also provide “*on‐teeth data*”, relating to the timestamps that the mouthguard is coupled to the teeth via an infrared sensor [16]. The on‐teeth data were used to establish the dates and times when an iMG was worn for each Super League team.

#### 2.2.2 GPS to Determine Training Exposure

GPS data is routinely collected by Super League teams for training and matches by a member of the sport science support staff within Super League teams [17, 18]. Staff identify periods of activity which are then manually created in proprietary software (Catapult OpenField, Catapult Sports, Melbourne, Australia) to reflect the start, end, and duration of drills within the session. The date (dd/mm/yyyy) and the staff‐identified start and end times (hh:mm:ss) of pitch‐based team training sessions, when iMGs were worn by players, were obtained to determine the overall duration of the training sessions.

### 2.3 Data Collection

#### 2.3.1 Instrumented Mouthguards (iMGs) to Measure Head Acceleration Events

The HAE and *on‐teeth data* were accessed through Prevent Biometrics' portal. All HAEs and *on‐teeth data* from 1 week prior to the start of Round 1 of fixtures until the end of Round 27 during the 2024 Super League season were accessed.

#### 2.3.2 GPS to Determine Player Training Session Exposure

GPS data were accessed and downloaded through Catapult's proprietary application programming interface (API), as outlined in previous research [19, 20]. All GPS periods of activity occurring on a date where *on‐teeth data* were present were downloaded (number of team training sessions accessed = 301). The session times were defined as the start timestamp of the first drill of the session to the end timestamp for the last drill of the session. All drills that reflected individual and rehabilitation sessions by name were removed from the dataset.

#### 2.3.3 Data Synchronization

To quantify HAE incidence per player‐training session, synchronization of data from both GPS and iMGs was required. The time alignment of session start and end times from GPS and SAE data was performed in R Studio (R Studio version 2023.12.1; R version 4.3.3). The *on‐teeth data* was time‐synchronized with the GPS to ensure that the iMG was worn between the start and end times of the pitch‐based session, and not at another time during the day (e.g., during indoor wrestle sessions). Following the synchronization, the *on‐teeth data* were visualized alongside the start and end times of individual drills within the session to allow for visual inspection of the data and identify periods where the iMG was not securely worn. This was conducted by the lead researcher during data analysis. Player‐sessions were excluded if *on‐teeth data* were deemed insufficient, defined as no clear indication that the iMG was worn either continuously or repeatedly during at least one drill within the session. This process led to the removal of 158 team sessions and 121 player sessions; examples of included and excluded player sessions are shown in Figure S1.

#### 2.3.4 Data Extraction

Following data synchronization, all HAEs occurring during the synchronized training sessions were then extracted. For the training dataset, this was achieved by extracting all SAE data that occurred between the specified start and end times accessed through GPS data. Count data were extracted for: (1) all recorded HAEs per session, (2) all HAEs exceeding PLA thresholds of 10, 25, 40, 55, and 70 *g*, and (3) all HAEs exceeding PAA thresholds of 1000, 2000, 3000, 4000, and 5000 rad⋅s^−2^ as per previous research in rugby league [7].

#### 2.3.5 Match Data

For the match data, a cross‐correlation approach [21] was employed to synchronize iMG data (HAE and *on‐teeth data*) to match event data (StatsPerform, Chicago, IL, USA) containing timestamps for every contact event (tackles and ball carries) to ensure players wore iMGs during tackle involvements. Only player matches whereby 90% of the tackle involvements occurred during periods where *on‐teeth data* was present were included in the dataset as per previous research [7, 14, 16]. Only players whose data were included in the training dataset were included in the match dataset.

### 2.4 Statistical Analysis

All statistical analysis was completed in R studio (Version 2024.12.1, R Foundation for Statistical Computing, Vienna, Austria). Incidence rates of HAEs during player‐training sessions and player‐matches where iMGs were worn were estimated using a Generalized Linear Mixed Model with Poisson distribution and log link function via *glmmTMB* (Version 1.1.9) [22]. The dependent variable was HAE count with session time and activity type (training or match) as fixed effects, along with *player ID* and *team ID* as random effects. A separate model was used to estimate HAE incidence rates at various PLA magnitude thresholds; this model contained HAE count for a given threshold as the outcome measure, session time and activity type (training or match), and PLA threshold as fixed effects, and *player ID* and *team ID* as random effects. Session incidence rates were calculated per hour of training and match‐play. Incidence rate ratios (IRR) with 95% confidence intervals (CI) were calculated to compare the HAE incidence rates during training and match‐play for forwards and backs via *emmeans* (version 1.10.3) [23]. Differences were deemed substantially different when the 95% CIs of the IRRs did not cross the null value of 1.

## 3 Results

### 3.1 HAE Count and Incidence During Training and Matches

**FIGURE 2:**
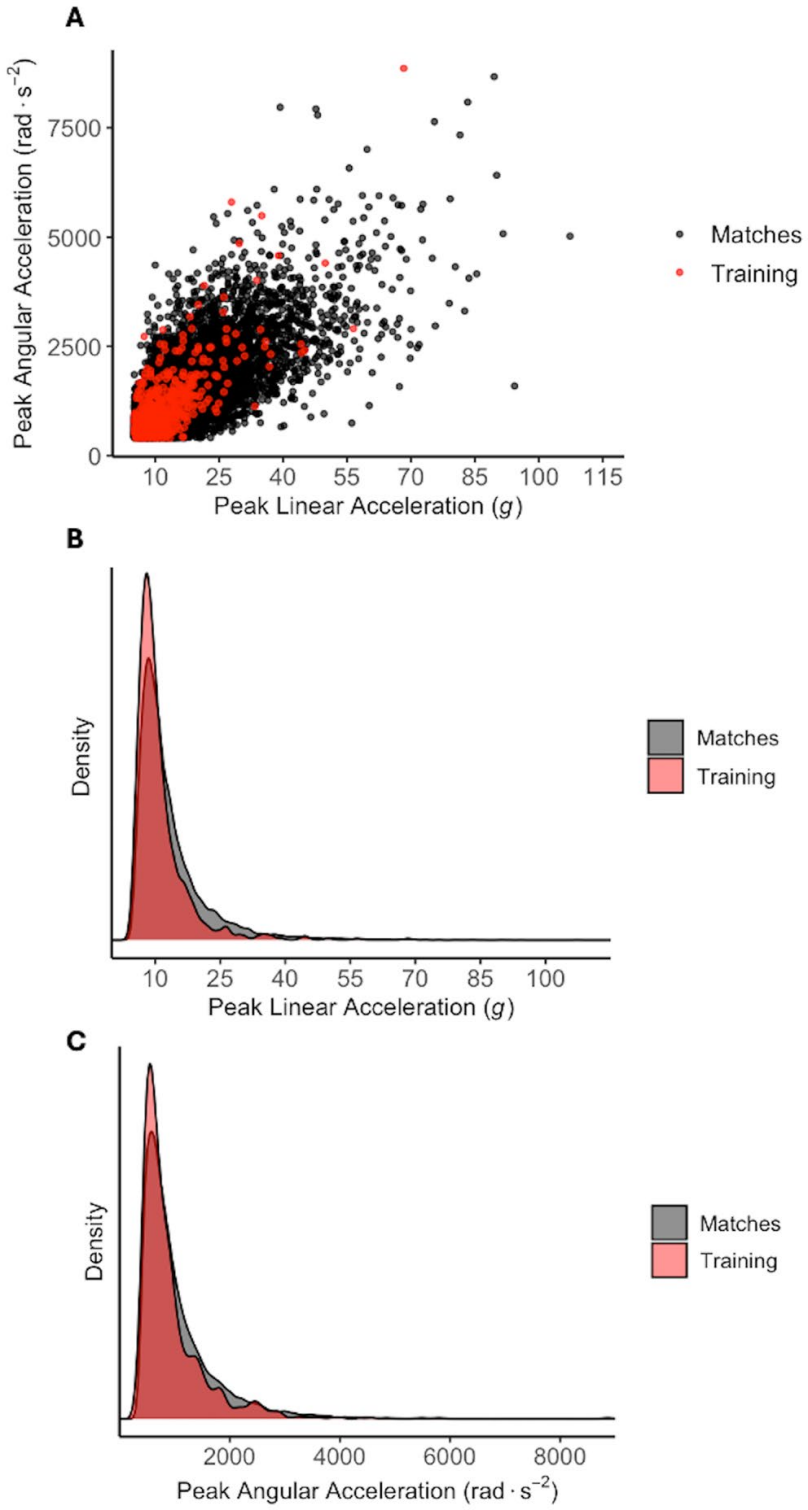
The PLA (*g*) and PAA rad⋅s^−2^ of each 814 HAEs during 468 player‐training sessions and 18043 HAEs during 665 player matches where an instrumented mouthguard was worn (A) and the distribution of HAE magnitude for PLA (B) and PAA (C).

A total of 814 HAEs were recorded across 538 player‐training hours (mean session time = 69 ± 17 min, mean time on‐teeth = 29 ± 17 min), while 18 043 HAEs were recorded across 653 player‐match hours (mean playing time = 58 ± 22 min) (Figure 2a). Figure 2b,c illustrate that the distribution of HAEs by magnitude was greater in training than in matches for values below 15 g and 1000 rad·s^−2^, indicating a tendency to record lower magnitude HAEs in training compared to matches. The median (IQR) PLA and PAA for training vs. matches was 9.3 *g* (7.6–12.4 *g*) vs. 10.9 *g* (8.4–15.4 *g*) and 722 rad⋅s^−2^ (551–1060 rads^2^) vs. 836 rad⋅s^−2^ (606–1261 rad⋅s^−2^) respectively. Some higher magnitude HAEs were recorded during training (Figure 2a), including 30 HAEs exceeding 25 *g*, 6 exceeding 40 *g*, and 2 exceeding 55 *g*. The 99th percentile PLA and PAA values were 36.8 *g* and 3896 rad⋅s^−2^ for training and 45.9 *g* and 3738 rad⋅s^−2^ for matches.

**FIGURE 3:**
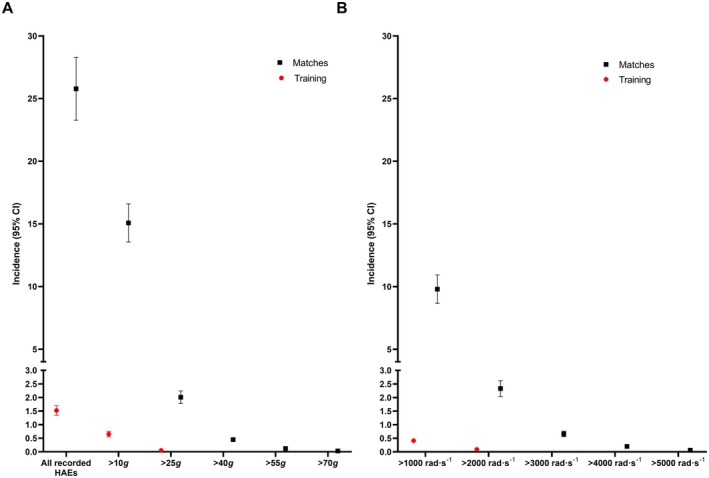
The mean incidence and 95% CIs of HAEs per hour of training and match‐play for (A) PLA and (B) PAA thresholds. All recorded HAEs are > 5 *g* and > 400 rad⋅s^−2^.

Figure 3 shows the estimated HAE incidence (95% CI) per hour of training and match‐play. Comparisons of HAE incidence in training and matches are shown in Table 1. The incidence (training vs. match) for HAEs exceeding the lowest magnitude threshold (5 *g* and 400 rad⋅s^−2^) was 1.52 (1.34–1.70) vs. 25.78 (23.28–28.27) HAEs per player‐hour, 0.65 (0.51–0.69) vs. 15.08 (13.55–16.60) for HAEs > 10 *g*, and 0.04 (0.02–0.06) vs. 2.01 (1.79–2.24) for HAEs > 25 *g*. HAE incidence rates were significantly greater during matches than training, with incidence rates being ~17 times greater for all recorded HAEs, ~23 times greater for HAEs with a PLA magnitude > 10 *g*, and ~40 times greater for HAEs with a PLA magnitude > 25 *g* (Table 1).

**TABLE 1:** The HAE incidence rate ratios (IRR) for match‐play vs. training.

Comparison		Player sessions (*n*)	SAEs (*n*)	IRR (95% CI)
All recorded HAEs (> 5 *g* and > 400 rad⋅s^−2^)	Training	468	814	1 (Reference value)
	Match	665	18 043	16.96 (14.92–19.01)^a^
PLA (> 10 *g*)	Training	468	355	1 (Reference value)
	Match	665	10 616	23.12 (21.03–25.20)^a^
PLA (> 25 *g*)	Training	468	30	1 (Reference value)
	Match	665	10 616	40.41 (37.96–42.86)^a^
PAA (> 1000 rad⋅s^−2^)	Training	468	228	1 (Reference value)
	Match	665	6953	23.85 (21.74–25.97)^a^
PAA (> 2000 rad⋅s^−2^)	Training	468	52	1 (Reference value)
	Match	665	6953	26.25 (23.95–28.56)^a^

*Note:* The incidence rates for > 40 *g*, > 55 *g* and 70 *g*, along with > 3000 rad⋅s^−2^, > 4000 rad⋅s^−2^, > 5000 rad⋅s^−2^ HAEs during training were < 0.01, therefore comparisons were not made.

^a^ Indicates substantial difference as 95% CIs did not cross the null value of 1.

Similarly, for > 1000 rad⋅s^−2^ HAEs, the incidence during training and match‐play was 0.41 (0.33–0.48) vs. 9.80 (8.67–10.93) and 0.09 (0.06–0.12) vs. 2.33 (2.04–2.61) for > 2000 rad⋅s^−2^. The incidence for > 40 *g*, > 55 *g*, and > 70 *g*, along with > 3000 rad⋅s^−2^, > 4000 rad⋅s^−2^, > 5000 rad⋅s^−2^ HAEs during training was all < 0.01 per player hour; therefore, they are not included in the figure. HAE incidence rates for HAEs with a PAA > 1000 rad⋅s^−2^ and > 2000 rad⋅s^−2^ are ~24 and ~26 times greater during matches, respectively, when compared to training.

No substantial differences in the incidence rate of HAEs were found between forwards and backs during training and matches. Positional data are presented in Figure S2 and Table S1.

### 3.2 Proportion of Player‐Training Sessions and Player Matches Where HAEs Occur

**FIGURE 4:**
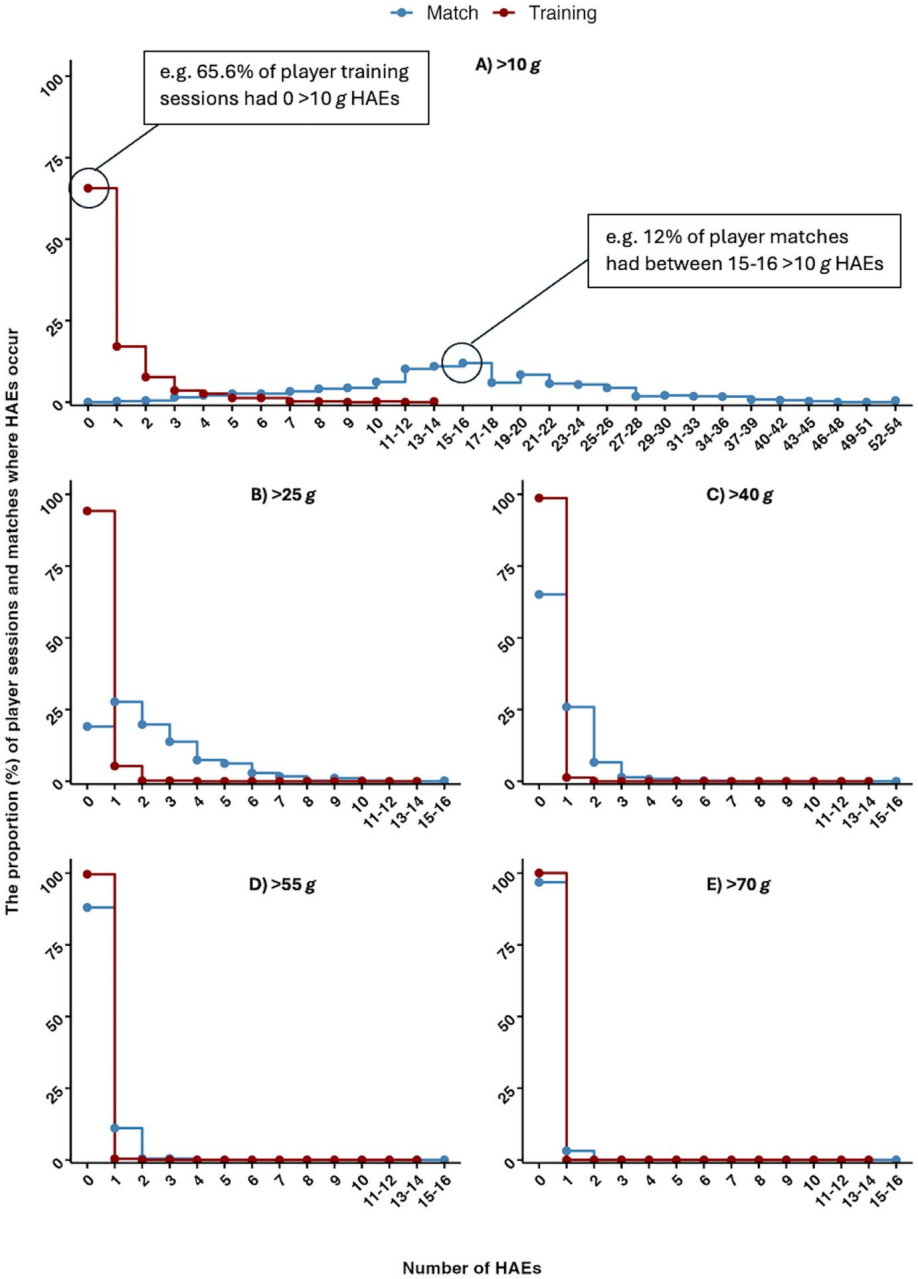
The proportion of player‐training sessions (total *n* = 468) and player matches (total *n* = 665) where HAEs with a PLA (A) > 10 *g*, (B) > 25 *g*, (C) > 40 *g*, (D) > 55 *g*, (E) > 70 *g* occur, and the number of HAEs occurring in a single session, and the number of HAEs exceeding each threshold occurring in a single player‐training session. On graph labels are included for illustrative purposes.

**FIGURE 5:**
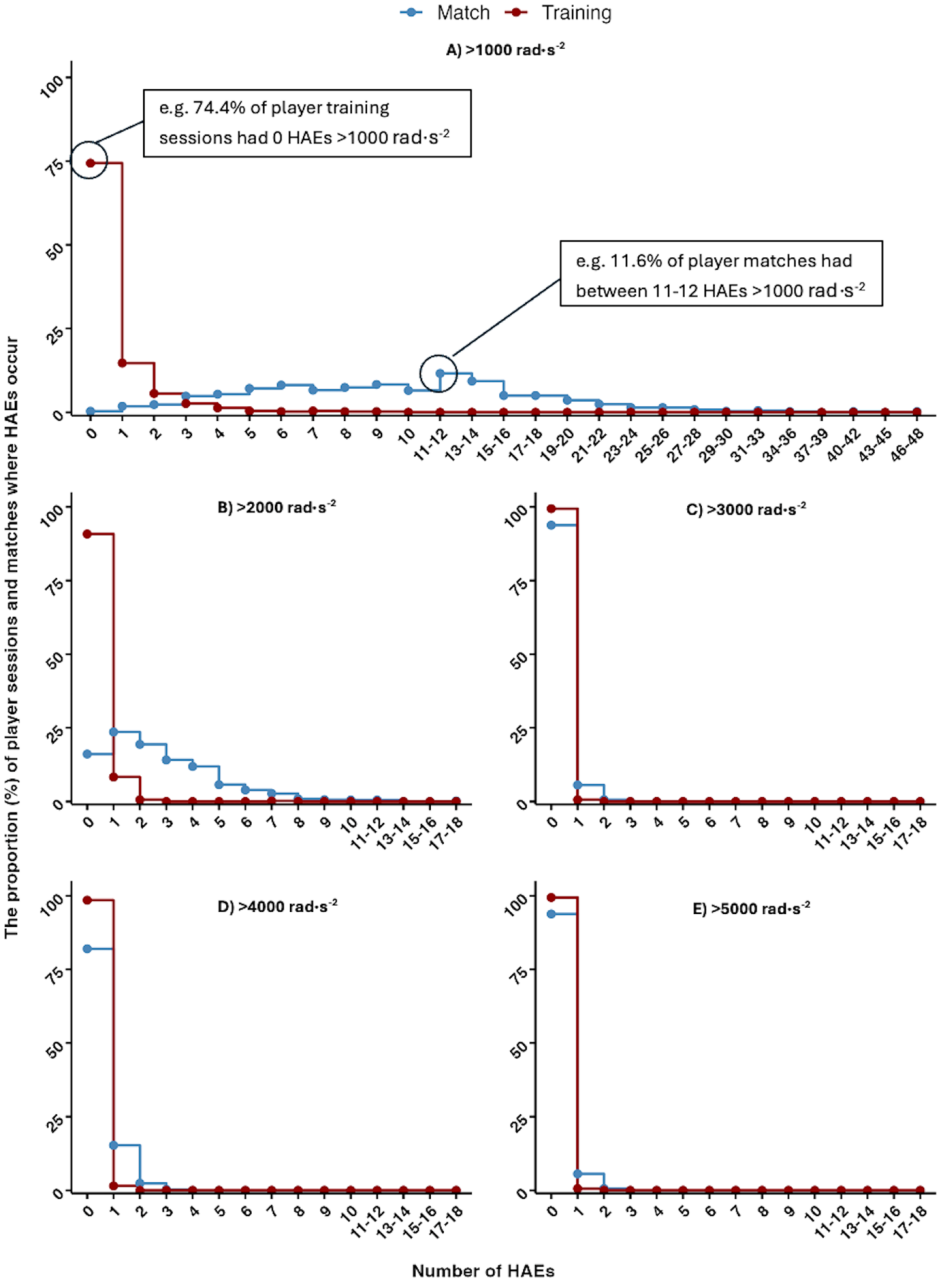
The proportion of player‐training sessions (total *n* = 468) and player‐matches (total *n* = 665) where HAEs with a PAA (A) > 1000 rad⋅s^−2^, (B) > 2000 rad⋅s^−2^, (C) > 3000 rad⋅s^−2^, (D) > 4000 rad⋅s^−2^, (E) > 5000 rad⋅s^−2^ occur, and the number of HAEs occurring in a single session, and the number of HAEs exceeding each threshold occurring in a single player‐training session. On graph labels are included for illustrative purposes.

The proportion of player‐training sessions where the number of HAEs exceeded a PLA magnitude of > 10 *g* to > 70 *g* and PAA magnitudes of > 1000 rad × s^−2^ to > 5000 rad × s^−2^ is shown in Figures 4 and 5 respectively. During the 468 player‐training sessions, 65.6% (*n* = 307) of player‐training sessions had no HAEs > 10 *g*. All player‐matches had one or more HAEs > 10 *g*. The highest number of HAEs > 10 *g* within a single player‐training session was 13; comparatively, 13 or more HAEs occurred in 62.4% of player‐matches. The highest number of HAEs > 10 *g* in a single player‐match was 53 HAEs. At least one HAE > 25 *g* was recorded in 5.6% (*n* = 27) player‐training sessions, while 80.9% of player‐matches recorded one or more HAEs > 25 *g*.

## 4 Discussion

This study aimed to quantify and describe the incidence of HAEs during pitch‐based training and to compare it with match‐play in professional male rugby league players. Training HAE incidence rate across all threshold ranges was lower than match‐play. The HAE incidence rate was 17 times greater in matches compared to training for all recorded HAEs, increasing to ~23 times and ~40 times greater for HAEs > 10 *g* and > 25 *g*. Despite this, higher magnitude HAEs did occur in training; the PLA and PAA values for the 99th percentile of HAEs were 36.8 *g* and 3896 rad⋅s^−2^ respectively for training, although this was lower than matches (45.9 *g* and 3738 rad⋅s^−2^). Most player‐training sessions had no HAEs > 10 *g* (65.6%), compared to match‐play where all player‐matches had one or more HAEs > 10 *g*, and in matches more than ten HAEs > 10 *g* were recorded for the majority of player‐matches (72.2%). Additionally, most player‐matches had at least one HAE > 25 *g* (80.9%), compared to training where only 5.8% of player‐training sessions had one or more HAEs > 25 *g*. Therefore, to reduce HAEs in training, targeting the small proportion of player‐training sessions which result in higher magnitude HAEs would be required, given the low overall rate. In comparison, given the higher HAE rates in matches, this may be a more beneficial target for HAE reduction interventions.

### 4.1 HAE Incidence During Training and Matches

Collectively, these values show that the incidence of HAEs during pitch‐based training was low both in incidence rate and magnitude. However, higher‐magnitude HAEs did occur, albeit rarely; therefore, studies should establish the player events or training events associated with these HAEs.

This is the first study to investigate the HAE incidence rates in rugby league training, as well as match‐play. Overall, the number of HAEs that occurred during training was relatively low, and HAEs with a higher PLA and PAA are rare. The incidence rates in the present study were slightly lower overall than research in rugby union [14, 24]. Roe et al. [14] reported 2.17 and 3.88 HAEs per player‐hour for backs and forwards respectively for all recorded HAEs (> 5 *g* and > 400 rad⋅s^−2^), and 0.12 and 0.27 for HAEs exceeding a PLA magnitude of > 25 *g*. These values are slightly greater than the 1.52 (1.34–1.70) HAEs per player‐hour observed in this study. A possible explanation may be the different contact demands or training practices between rugby league and union, although this is unknown. For example, in rugby league, indoor wrestle training forms a significant portion of contact training [11], and was not included in the present study. This potentially impacts the duration and intensity of contact seen during pitch‐based rugby league training and therefore HAE incidence. The low HAE exposure found within the present study is in line with previous research quantifying training injuries within rugby league, reporting low contact injury (Incidence [95% CI] 0.41 [0.30–0.54]) and head injury (Incidence [95% CI] 0.25 [0.17–0.37]) incidence within training [4].

A clear difference in HAE incidence rates between training and match‐play was identified. The incidence rate of HAEs > 5 g and 400 rad⋅s^−2^ was ~17 times greater during match‐play compared to training, and 23 and 24 times greater for > 10 *g* and > 1000 rad⋅s^−2^. This difference was even greater at higher magnitude HAEs (e.g., 40 and 26 times greater for > 25 *g* and 2000 rad⋅s^−2^ HAEs; Figure 2, Table 1), which was consistent for both forwards and backs (Figure S2). One potential reason for this difference is that teams have reported that on average they spend less than 30 min per week performing full‐contact training [11]. Contact training may be spread throughout the training week into multiple sessions, meaning the actual exposure to full‐contact training per session may be low. The present study also only assessed pitch‐based training sessions where iMGs were worn, so it is possible that HAEs of greater magnitudes do occur either in non‐pitch‐based sessions (e.g., gym‐based wrestle sessions) or by players who did not wear an iMG during training. Nonetheless, these findings are consistent with those in rugby union whereby the incidence rate of HAE events of all magnitudes is substantially lower during training compared to match‐play [14]. The assumption is that players will wear iMGs for the highest level of contact for dental protection; therefore, the HAEs and rates reported in this study may be from the highest level of contact.

### 4.2 Proportion of Training Sessions and Matches Where HAEs Occur

The findings of the study show that HAEs during pitch‐based rugby league training are not a common occurrence within a single session, with most player sessions accumulating zero HAEs > 10 *g*. Similarly, it is uncommon for players to experience multiple HAEs within a single session, with only ~18% and ~12% of player sessions containing two or more HAEs with a magnitude of > 10 *g* or 1000 rad⋅s^−2^. This is in contrast to match‐play, where all player matches had at least one > 10 *g* HAE, and most player matches had more than ten > 10 *g* HAEs. In addition, higher magnitudes of HAEs are more common, with 81% and 35% of matches having at least one > 25 *g* and 40 *g* HAE. Overall, this shows that it is likely that the accumulation of HAEs is influenced more by match‐play than training. To reduce HAEs in training, further research is required to understand which activities result in a player experiencing > 10 *g* HAEs, as well as the higher magnitude HAEs. Whilst rare, targeting these activities may reduce HAE exposure in training, alongside broader contact training guidelines [11], which exist. However, it is possible that further manipulation of contact training in an attempt to reduce already low HAE incidence could leave players unprepared for the physical and technical contact demands of match‐play [11]. Therefore, focusing on matches may allow for the biggest impact in reducing HAEs, which have been modeled for specific playing positions [6].

### 4.3 Limitations

The primary limitation of this study is that the HAE incidence has been estimated based on the assumption that players wear iMGs during pitch‐based contact training [24] thus HAEs are unlikely to occur elsewhere in the week. This assumption also involves players only wearing mouthguards during contact, leading to potentially low time‐on‐teeth (mean = 29 ± 17 min). The method used within the present study means it is not possible to provide any contextual information regarding HAEs in training (e.g., tackle or ball‐carry, or training drill where they occur) along with whether all players were present for the whole session, leading to further assumptions. However, accessing this information would require each training session to be video recorded with accurate timestamps and the research team being granted access. The present study contains no information regarding the injury status or outcomes (e.g., concussion) of the recorded HAEs. A second limitation is the potential underestimation of lower magnitude HAEs (< 25 *g*) as a result of the potential linear acceleration bias. This is due to a discrepancy in the linear acceleration measured at the iMG location compared to the resultant values transformed at the head centre of gravity. However, comparisons between training and match‐play remain unaffected as it is assumed the bias affects both equally [25, 26]. Additionally, in the absence of clinical thresholds, arbitrary thresholds of > 10 and > 25 g have been used within the study, consistent with previous published research [7]. Finally, the present study only assessed pitch‐based training sessions where both GPS and iMGs were worn; this potentially limits the training types assessed, for example, wrestle training which may be performed indoors, thus underestimating players' actual weekly exposure.

### 4.4 Perspective

Overall, HAEs in rugby league pitch‐based training where players wear iMGs are relatively rare, with over 65% of player sessions having no HAEs above 10 *g*. Similarly, mean incidence rates for HAEs above 10 *g* are below one HAE per player‐hour of training, and substantially lower than incidence rates seen in match‐play. Despite low incidence rates, some higher magnitude HAEs did occur in training, and some player sessions accumulate multiple HAEs. As such, practitioners and governing bodies should continue to monitor training practices and HAEs in training to ensure players are not exposed unnecessarily to HAEs whilst also allowing for sufficient contact training exposure to prepare players for the physical and technical contact demands of match‐play. Reducing unnecessary HAEs throughout a player's playing career may reduce the risk of negative brain health outcomes in later life.

## Conflicts of Interest

The study was undertaken as part of a funded research study (Tackle and Contact, Kinematics, Load and Exposure; TaCKLE project) ‐ with funding provided by the Rugby Football League. J.P. PhD is part‐funded by the Rugby Football Leavgue. C.O., J.T. and N.C. Fellowships are part‐funded by the Rugby Football League. G.P. is employed by the Rugby Football League. D.V. and B.J. are employed by the Rugby Football League in a consultancy capacity. B.J. is employed by PREM Rugby in a consultancy capacity. K.S. is employed by the Rugby Football Union.

## Supporting information


**Appendix S1:** sms70156‐sup‐0001‐AppendixS1.docx.

## Data Availability

Research data is not shared.
