# Supplementary material for: The Incidence of Head Acceleration Events During Pitch‐Based Training and Match Play in Professional Men's Rugby League
Source: Scand J Med Sci Sports. 2025 Nov 8;35(11):e70156. doi: 10.1111/sms.70156 (PMC12595519; doi:10.1111/sms.70156)
Supplement: Supplementary file 1 — Appendix S1: sms70156‐sup‐0001‐AppendixS1.docx. [file SMS-35-e70156-s001.docx]

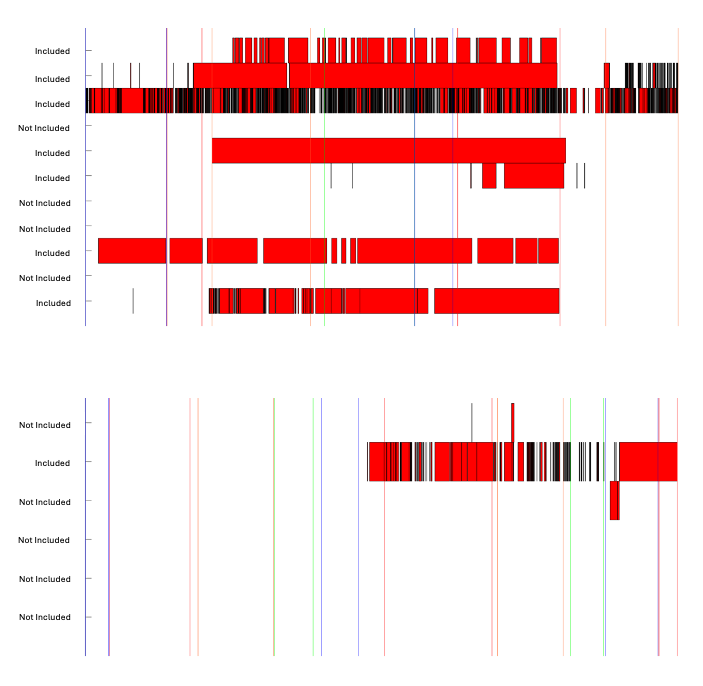


Supplementary Figure 1. Examples of on-teeth data plotted alongside session and drill start/end times, used to determine player session inclusion. Player sessions with clear indication that the iMG was worn either continuously or repeatedly during at least one drill within the session were included within the analysis. Player sessions with missing or inconsistent data were excluded.


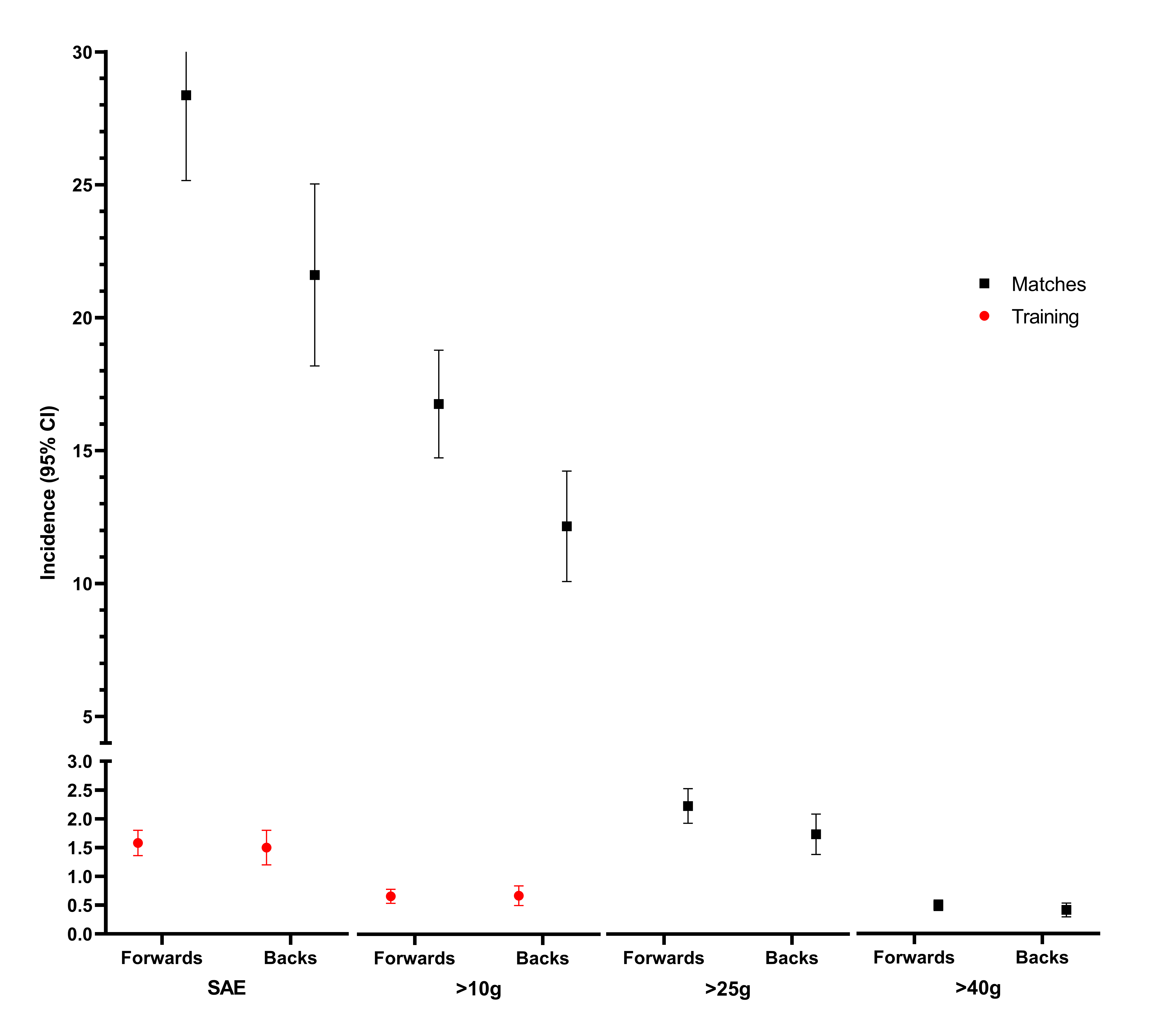


Supplementary Figure 2. The mean incidence and 95% CIs of HAEs for forwards and backs per hour of training and match-play for A) PLA and B) PAA thresholds. SAE are >5 *g* and >400 rad×s^-2^.

| Supplementary Table 1. The HAE incidence rate ratios (IRR) for forwards vs backs during match-play and training | | | | |
| --- | --- | --- | --- | --- |
| **Comparison** | | **Player Sessions (n)** | **SAEs (n)** | **IRR (95% CI)** |
| Training All recorded HAEs (>5 *g* and >400 rad×s^-2^) | Backs | 122 | 206 | 1 (Reference Value) |
|  | Forwards | 346 | 608 | 1.06 (-1.16 - 3.28) |
| Match All recorded HAEs (>5 *g* and >400 rad×s^-2^) | Backs | 197 | 4606 | 1 (Reference Value) |
|  | Forwards | 468 | 13437 | 1.31 (-0.85 - 3.48) |
| Training  (>10g) | Backs | 122 | 98 | 1 (Reference Value) |
|  | Forwards | 346 | 257 | 0.99 (-1.31 - 3.28) |
| Match  (>10g) | Backs | 197 | 2795 | 1 (Reference Value) |
|  | Forwards | 468 | 7821 | 1.38 (-0.80 - 3.56) |
| Training  (>1000 rad×s^-2^) | Backs | 122 | 71 | 1 (Reference Value) |
|  | Forwards | 346 | 157 | 0.87 (-1.49 - 3.24) |
| Match  (>1000 rad×s^-2^) | Backs | 197 | 1770 | 1 (Reference Value) |
|  | Forwards | 468 | 5183 | 1.34 (-0.88 - 3.55) |
